# Tris(1,10-phenanthroline-κ^2^ *N*,*N*′)nickel(II) hexa­oxido-μ-peroxido-disulfate­(VI) *N*,*N*-dimethyl­formamide disolvate monohydrate

**DOI:** 10.1107/S1600536812050775

**Published:** 2012-12-22

**Authors:** Miguel Angel Harvey, Sebastián Suarez, Fabio Doctorovich, Ricardo Baggio

**Affiliations:** a Facultad de Ciencias Naturales, Universidad Nacional de la Patagonia S.J.B., Sede Trelew, 9100 Trelew, Chubut, Argentina; b CenPat, CONICET, 9120 Puerto Madryn, Chubut, Argentina; c Departamento de Química Inorgánica, Analítica y Química, Física/INQUIMAE–CONICET, Facultad de Ciencias Exactas y Naturales, Universidad de Buenos Aires, Buenos Aires, Argentina; d Gerencia de Investigación y Aplicaciones, Centro Atómico Constituyentes, Comisión Nacional de Energía Atómica, Buenos Aires, Argentina

## Abstract

The asymmetric unit of the title complex, [Ni(C_12_H_8_N_2_)_3_]S_2_O_8_·2C_3_H_7_NO·H_2_O, consists of a complex [Ni(phen)_3_]^2+^ cation and one isolated pds anion, with two DMF mol­ecules and one water mol­ecule as solvates (where phen is 1,10-phenanthroline, pds is the hexa­oxido-μ-peroxoido-di­sulf­ate dianion and DMF is dimethyl­formamide). The [Ni(phen)_3_]^2+^ cation is regular, with an almost ideal Ni^II^ bond-valence sum of 2.07 v.u. The group, as well as the water solvent mol­ecule, are well behaved in terms of crystallographic order, but the remaining three mol­ecules in the structure display different kinds of disorder, *viz.* the two DMF mol­ecules mimic a twofold splitting and the pds anion has both S atoms clamped at well-determined positions but with a not-too-well-defined central part. These peculiar behaviours are a consequence of the hydrogen-bonding inter­actions: the outermost SO_3_ parts of the pds anion are heavily connected to the complex cations *via* C—H⋯O hydrogen bonding, generating an [Ni(phen)_3_]pds network and providing for the stability of the terminal pds sites. Also, the water solvent mol­ecule is strongly bound to the structure (being a donor of two strong bonds and an acceptor of one) and is accordingly perfectly ordered. The peroxide O atoms in the pds middle region, instead, appear as much less restrained into their sites, which may explain their tendency to disorder. The cation–anion network leaves large embedded holes, amounting to about 28% of the total crystal volume, which are occupied by the DMF mol­ecules. The latter are weakly inter­acting with the rest of the structure, which renders them much more labile and, accordingly, prone to disorder.

## Related literature

For information on structures with coordinated pds, see: Youngme *et al.* (2007 ▶); Manson *et al.* (2009 ▶); Harrison & Hathaway (1980 ▶); Blackman *et al.* (1991 ▶); Harvey *et al.* (2011 ▶) and references therein. For examples of structurers with non-coordinating pds groups, see Baffert *et al.* (2009 ▶); Harvey *et al.* (2004 ▶, 2005 ▶); Youngme *et al.* (2008 ▶); Singh *et al.* (2009 ▶). For details of bond-valence analysis and the vector bond-valence model, see: Brown & Altermatt (1985 ▶) and Harvey *et al.* (2006 ▶), respectively.
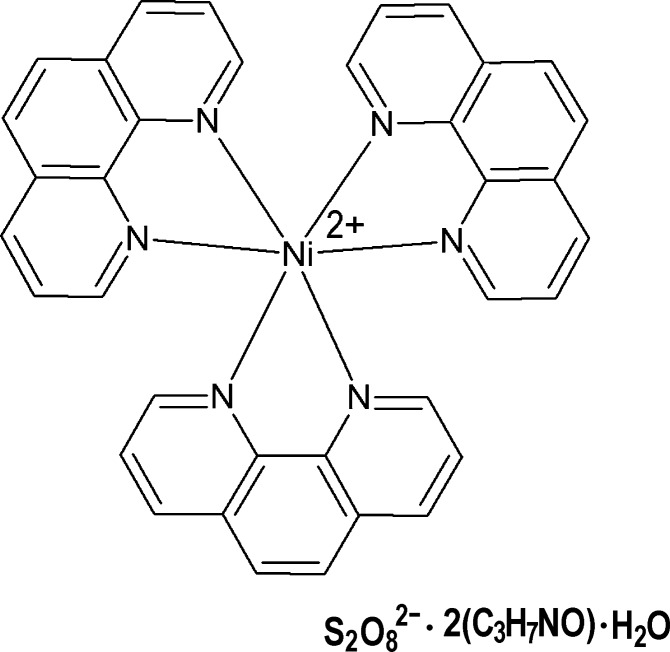


## Experimental

### 

#### Crystal data

[Ni(C_12_H_8_N_2_)_3_](S_2_O_8_)·2C_3_H_7_NO·H_2_O
*M* *_r_* = 955.65
Triclinic, 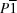

*a* = 10.4832 (3) Å
*b* = 12.2221 (4) Å
*c* = 18.0044 (6) Å
α = 79.691 (3)°
β = 76.725 (3)°
γ = 76.190 (3)°
*V* = 2161.41 (12) Å^3^
*Z* = 2
Mo *K*α radiation
μ = 0.62 mm^−1^
*T* = 294 K
0.18 × 0.11 × 0.11 mm

#### Data collection

Oxford Diffraction Gemini CCD S Ultra diffractometer
Absorption correction: multi-scan (*CrysAlis PRO*; Oxford Diffraction, 2009 ▶) *T*_min_ = 0.945, *T*_max_ = 0.952
31646 measured reflections
10070 independent reflections
6165 reflections with *I* > 2σ(*I*)
*R*_int_ = 0.041

#### Refinement

*R*[*F* ^2^ > 2σ(*F* ^2^)] = 0.056
*wR*(*F* ^2^) = 0.170
*S* = 1.04
10070 reflections
647 parameters
246 restraints
H atoms treated by a mixture of independent and constrained refinement
Δρ_max_ = 0.65 e Å^−3^
Δρ_min_ = −0.73 e Å^−3^

### 

Data collection: *CrysAlis PRO* (Oxford Diffraction, 2009 ▶); cell refinement: *CrysAlis PRO*; data reduction: *CrysAlis PRO*; program(s) used to solve structure: *SHELXS97* (Sheldrick, 2008 ▶); program(s) used to refine structure: *SHELXL97* (Sheldrick, 2008 ▶); molecular graphics: *SHELXTL* (Sheldrick, 2008 ▶); software used to prepare material for publication: *SHELXL97* and *PLATON* (Spek, 2009 ▶).

## Supplementary Material

Crystal structure: contains datablock(s) I, global. DOI: 10.1107/S1600536812050775/br2216sup1.cif

Structure factors: contains datablock(s) I. DOI: 10.1107/S1600536812050775/br2216Isup2.hkl

Additional supplementary materials:  crystallographic information; 3D view; checkCIF report

## Figures and Tables

**Table 1:** Hydrogen-bond geometry (Å, °)

*D*—H⋯*A*	*D*—H	H⋯*A*	*D*⋯*A*	*D*—H⋯*A*
O1*W*—H1*WA*⋯O6^i^	0.85 (5)	2.02 (6)	2.839 (7)	160 (10)
O1*W*—H1*WB*⋯O1*D*′^i^	0.85 (7)	1.90 (7)	2.668 (10)	149 (7)
C3*B*—H3*B*⋯O1*W* ^ii^	0.93	2.54	3.305 (8)	139
C1*B*—H1*B*⋯O3^ii^	0.93	2.55	3.192 (6)	126
C3*A*—H3*A*⋯O8	0.93	2.59	3.271 (6)	130
C3*C*—H3*C*⋯O1^iii^	0.93	2.43	3.337 (6)	164
C5*A*—H5*A*⋯O3	0.93	2.58	3.505 (7)	170
C5*C*—H5*C*⋯O2^iii^	0.93	2.53	3.365 (7)	150
C6*B*—H6*B*⋯O1^i^	0.93	2.53	3.434 (5)	163
C6*C*—H6*C*⋯O2^iv^	0.93	2.56	3.409 (6)	151
C8*C*—H8*C*⋯O3^iv^	0.93	2.30	3.197 (6)	162
C10*A*—H10*A*⋯O8^v^	0.93	2.48	3.220 (6)	137
C10*C*—H10*C*⋯O1*E*′	0.93	2.59	3.228 (19)	126

Symmetry codes: (i) 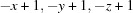; (ii) 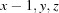; (iii) 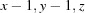; (iv) 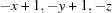; (v) 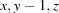.

**Fig. 1.**
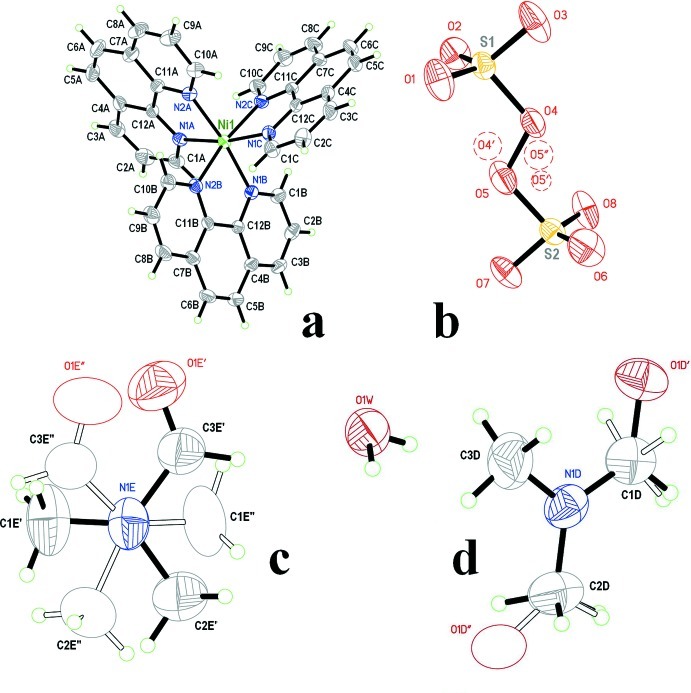
Ellipsoid plot of the different constituents of (I), drawn at a 30% displacement factor level. (a): The Ni(phen)3 nucleus. (b): The S2O82- anion. Note the minoritarian S fractions, around O4 and O5. (c) and (d): the two disordered DMF molecules, with different pseudo two-fold symmetry, and the well behaved water solvate.

**Fig. 2.**
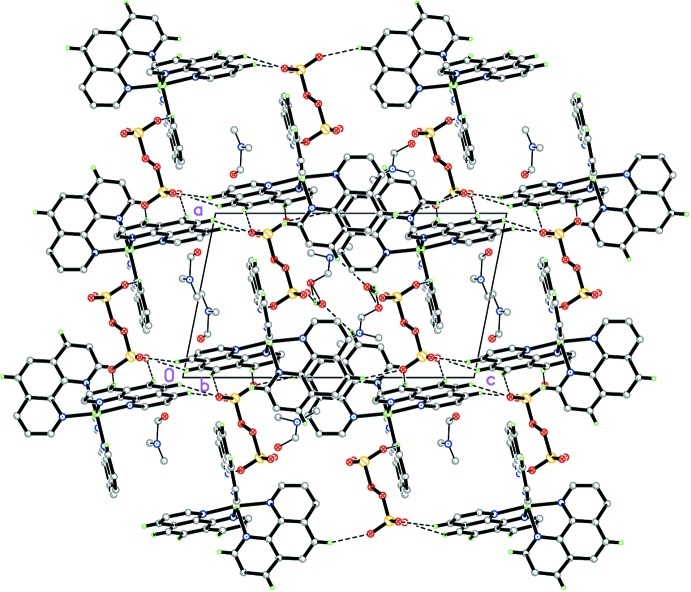
A packing view of (I) drawn down b and showing the way in which the cationic-anionic H-bonding network builds up. DMF units (in light lining) appear in the structural voids, with almost no connections to the rest. Only H atoms involved in H-bonding have been represented.

## supplementary crystallographic information

### Comment

The binding behavior of peroxodisulfate (pds) towards a number of transition metal metal cations (Cd(II), Hg(II), Cu(II), Mn(III), Zn(II), Ag(II)) has been well documentated in the literature Youngme *et al.*, 2007; Manson *et al.*, 2009., Blackman *et al.*, 1991; Harrison *et al.*, 1980; Harvey *et al.*, 2011, and references therein) but its rather elusive character as a ligand has also been evidenced in many other structures where the anion wouldn't coordinate, thus acting as a balancing counterion or, in occasions, just as a neutral co-crystallization agent in the form of peroxodisulfuric acid. Among the cations being reluctant towards pds coordination it must be mentioned the case of Cd (Harvey *et al.*, 2005); Co(III) (Singh *et al.*, 2009); Zn(II) (Harvey *et al.*, 2004), Cu(II) (Youngme *et al.*, 2008), Mn(IV) (Baffert *et al.*, 2009), and even the more stringent case of Ni, of which no crystal structure with pds had been reported up to date: in particular, all our previous experiments aimed to produce such a complex had so far been unsuccessful.

Therefore, we present herein the first Ni^II^-pds structure, where the anion did not enter into the Ni^II^ coordination sphere but behaves instead as a stabilizing counteranion: [Ni(phen)_3_]^2+^.(pds).2DMF.(H_2_O), where (phen: 1,10-phenanthroline; pds: peroxidisulfate dianion; DMF: dimethylformamide).

The asymmetric unit of the complex consists of a globular [Ni(phen)_3_]^2+^ nucleus (Fig 1a), one isolated pds anion, two DMF and one water molecules as solvates.

The [Ni(phen)_3_]^2+^ cationic centre is absolutely regular and does not differ from the more than 100 similar groups which appear in the v5.33 version of the CSD (Allen, 2002). The Bond Valence Sum for the Ni^II^ cation in the title compound (Brown and Altermatt, 1985) is almost ideal (2.07 v.u.), and the regularity in the NiN_6_ coordination sphere is shown by the tight range of similar parameters (d(Ni-N): 2.087 (3)-2.100 (3)Å; N-Ni-N cis angles: 79.04 (12)-79.71 (12)° (chelating); 92.79 (12)-96.46 (12)° (non-chelating); N-Ni-N trans angles: 172.06 (12)-170.20 (12)°), but it can perhaps be best assessed by the geometric disposition of the three Bond Valence Vectors associated to the three chelating phen ligands (for details, see Harvey *et al.*, 2006) which define an absolute planar array (sum of internal angles: 360.00°), and a theoretical (almost nil) resultant vector ( 0.017 v.u.). The cationic group as well as the water solvate are well behaved in terms of crystallographic order, but the remaining three groups in the structure display different kinds of disorder, as explained in detail in the refinement section, the two DMF mimicking different kinds of two-fold splitting (Figs. 1c,1d) with occupation factors of 0.546 (12)/0.454 (12) and 0.520 (12)/0.480 (12), respectively. In the case of pds this occurs in a more complicated fashion, having both S's "clamped" at two well determined positions (Fig 1b) and a not-so-well-defined central part (Occupation for O4,O5:0.641 (3)).

These peculiar behaviours may be better understood by inspection of Table 1, which gives the H-bonding interactions, and their representation in Fig 2. It is clearly seen therein that the outermost SO_3_ parts of the pds anion are strongly connected to the cationic centres via H-bonding (10 donors out of 13 correspond to these groups), generating a sort of stable [Ni(phen)_3_]-pds network and acting as a clamp for the terminal SO_3_ groups. The oxygens in the pds middle region, instead, are much less restrained to their sites, and this could explain some tendency to disorder. Similar mobility restrictions apply to the water solvate, donor of two strong bonds (Table 1, entries 1 and 2) and acceptor of one (3rd entry). On the other hand, the above cationic-anionic network leaves large embedded holes (about 28% of the total crystal volume, as calculated by PLATON, Spek, 2009). These holes are occupied by the DMF molecules (in light tracing in Fig 2). Analysis of the acceptors in Table 1 and inspection of Figure 2 reaveals that they hardly interact with the rest of the structure, being thus labile and, accordingly, prone to disorder.

### Experimental

The title compound was prepared by adding DMF to a solid, equimolar mixture of [Ni(CH_3_COO)_2_].4H_2_O, K_2_S_2_O_8_ and phen.H_2_O in such a way that phen final concentration was 0.500 M. Crystals suitable for X-ray diffraction developed in a few hours.

### Refinement

All C—H atoms were found in a difference map, but treated differently in refinement. Those attached to C were further idealized and finally allowed to ride. CH_3_ groups were also free to rotate. Water H's were refined with restrained d(O-H). In all cases displacement parameters were taken as *U*_iso_(H) = *X*×*U*_eq_(host) [d(*C*—*H*)_methyl_ = 0.96 A°, *X* = 1.5; d(C—H)_arom_ = 0.93 Å, *X* = 1.2; O - H = 0.85 (1)Å, X = 1.2].

A rather peculiar characteristic of the structure was its having the two DMF solvates as well as the pds anion disordered, all of them in different ways: in both DMF molecules the disorder mimics a two fold symmetry, with the pseudo two fold axis by force passing throuh the central N; in the case of moieties E (D) this occurs with the pseudo diad being perpendicular (parallel) to one of the to the two C(methyl)—N lines, Fig 1c (1d).

The case of the pds anion was not that clear cut, but interesting anyway: the molecule occupies in the crystal several, slightly offset positons, all of them with the S atoms "clamped" in the S1, S2 reported coordinates (No "ghosts" in their neighbourhood). The central oxygens O4 and O5, instead, presented a clear splitting which needed to be included in the model in order to have a proper refinement. The coresponding outermost minoritarian oxygens, however, could not be clearly disclosed and have to be accordingly disregarded. To compensate for this fact, atoms O1-O3, O6-O8 were given full occupancy. This procedure, fulfilled with some restraints in metrics and in displacement factors, allowed to reduce the R factor by more ~10%, and the s.u.'s for the O4, O5 coordinates in ~30%.

### Crystal data

**Table:**

[Ni(C_12_H_8_N_2_)_3_](S_2_O_8_)·2C_3_H_7_NO·H_2_O	*Z* = 2
*M**_r_* = 955.65	*F*(000) = 992
Triclinic, *P*1	*D*_x_ = 1.468 Mg m^−^^3^
Hall symbol: -P 1	Mo *K*α radiation, λ = 0.71073 Å
*a* = 10.4832 (3) Å	Cell parameters from 11752 reflections
*b* = 12.2221 (4) Å	θ = 3.7–28.8°
*c* = 18.0044 (6) Å	µ = 0.62 mm^−^^1^
α = 79.691 (3)°	*T* = 294 K
β = 76.725 (3)°	Block, light brown
γ = 76.190 (3)°	0.18 × 0.11 × 0.11 mm
*V* = 2161.41 (12) Å^3^	

### Data collection

**Table:**

Oxford Diffraction Gemini CCD S Ultra diffractometer	10070 independent reflections
Radiation source: fine-focus sealed tube	6165 reflections with *I* > 2σ(*I*)
Graphite monochromator	*R*_int_ = 0.041
ω scans, thick slices	θ_max_ = 28.9°, θ_min_ = 3.7°
Absorption correction: multi-scan (*CrysAlis PRO*; Oxford Diffraction, 2009)	*h* = −14→14
*T*_min_ = 0.945, *T*_max_ = 0.952	*k* = −15→16
31646 measured reflections	*l* = −24→24

### Refinement

**Table:**

Refinement on *F*^2^	Primary atom site location: structure-invariant direct methods
Least-squares matrix: full	Secondary atom site location: difference Fourier map
*R*[*F*^2^ > 2σ(*F*^2^)] = 0.056	Hydrogen site location: inferred from neighbouring sites
*wR*(*F*^2^) = 0.170	H atoms treated by a mixture of independent and constrained refinement
*S* = 1.04	*w* = 1/[σ^2^(*F*_o_^2^) + (0.096*P*)^2^] where *P* = (*F*_o_^2^ + 2*F*_c_^2^)/3
10070 reflections	(Δ/σ)_max_ = 0.007
647 parameters	Δρ_max_ = 0.65 e Å^−^^3^
246 restraints	Δρ_min_ = −0.73 e Å^−^^3^

### Special details

**Table:**

Geometry. All esds (except the esd in the dihedral angle between two l.s. planes) are estimated using the full covariance matrix. The cell esds are taken into account individually in the estimation of esds in distances, angles and torsion angles; correlations between esds in cell parameters are only used when they are defined by crystal symmetry. An approximate (isotropic) treatment of cell esds is used for estimating esds involving l.s. planes.

### Fractional atomic coordinates and isotropic or equivalent isotropic displacement parameters (Å^2^)

**Table:**

	*x*	*y*	*z*	*U*_iso_*/*U*_eq_	Occ. (<1)
Ni1	0.21814 (5)	0.29652 (4)	0.27142 (3)	0.04038 (18)	
N1A	0.3061 (3)	0.4386 (3)	0.25528 (18)	0.0449 (8)	
N2A	0.4160 (3)	0.2281 (3)	0.22251 (18)	0.0441 (8)	
C1A	0.2489 (5)	0.5435 (4)	0.2693 (3)	0.0564 (11)	
H1A	0.1571	0.5607	0.2880	0.068*	
C2A	0.3211 (6)	0.6290 (4)	0.2570 (3)	0.0707 (14)	
H2A	0.2781	0.7014	0.2690	0.085*	
C3A	0.4549 (6)	0.6067 (4)	0.2273 (3)	0.0678 (14)	
H3A	0.5037	0.6636	0.2191	0.081*	
C4A	0.5186 (5)	0.4977 (4)	0.2092 (2)	0.0552 (11)	
C5A	0.6573 (5)	0.4663 (5)	0.1758 (3)	0.0713 (14)	
H5A	0.7109	0.5198	0.1667	0.086*	
C6A	0.7116 (5)	0.3623 (5)	0.1573 (3)	0.0770 (16)	
H6A	0.8021	0.3451	0.1349	0.092*	
C7A	0.6338 (4)	0.2757 (4)	0.1711 (3)	0.0603 (12)	
C8A	0.6837 (5)	0.1661 (5)	0.1533 (3)	0.0751 (15)	
H8A	0.7735	0.1447	0.1307	0.090*	
C9A	0.6032 (5)	0.0890 (4)	0.1684 (3)	0.0722 (14)	
H9A	0.6366	0.0158	0.1554	0.087*	
C10A	0.4690 (5)	0.1224 (4)	0.2038 (3)	0.0576 (11)	
H10A	0.4143	0.0696	0.2150	0.069*	
C11A	0.4973 (4)	0.3038 (4)	0.2064 (2)	0.0457 (9)	
C12A	0.4382 (4)	0.4166 (3)	0.2246 (2)	0.0441 (9)	
N1B	0.0317 (3)	0.3791 (3)	0.32722 (18)	0.0429 (7)	
N2B	0.2391 (3)	0.2495 (3)	0.38604 (18)	0.0448 (8)	
C1B	−0.0697 (4)	0.4466 (4)	0.2978 (3)	0.0546 (11)	
H1B	−0.0617	0.4583	0.2446	0.066*	
C2B	−0.1862 (4)	0.5002 (4)	0.3420 (3)	0.0588 (12)	
H2B	−0.2541	0.5467	0.3187	0.071*	
C3B	−0.2005 (4)	0.4843 (4)	0.4201 (3)	0.0571 (11)	
H3B	−0.2777	0.5206	0.4505	0.068*	
C4B	−0.0975 (4)	0.4125 (4)	0.4541 (2)	0.0496 (10)	
C5B	−0.1039 (5)	0.3901 (4)	0.5355 (3)	0.0626 (12)	
H5B	−0.1807	0.4218	0.5685	0.075*	
C6B	0.0010 (5)	0.3229 (4)	0.5651 (2)	0.0597 (12)	
H6B	−0.0056	0.3088	0.6182	0.072*	
C7B	0.1210 (5)	0.2737 (4)	0.5163 (2)	0.0517 (10)	
C8B	0.2324 (5)	0.2047 (4)	0.5435 (3)	0.0658 (13)	
H8B	0.2316	0.1898	0.5961	0.079*	
C9B	0.3413 (5)	0.1598 (5)	0.4931 (3)	0.0715 (14)	
H9B	0.4154	0.1135	0.5108	0.086*	
C10B	0.3418 (5)	0.1835 (4)	0.4142 (3)	0.0598 (11)	
H10B	0.4171	0.1519	0.3801	0.072*	
C11B	0.1296 (4)	0.2931 (3)	0.4367 (2)	0.0415 (9)	
C12B	0.0175 (4)	0.3637 (3)	0.4050 (2)	0.0407 (8)	
N1C	0.1500 (3)	0.1494 (3)	0.26890 (19)	0.0473 (8)	
N2C	0.1687 (3)	0.3320 (3)	0.16241 (18)	0.0460 (8)	
C1C	0.1412 (5)	0.0598 (4)	0.3217 (3)	0.0600 (12)	
H1C	0.1629	0.0600	0.3690	0.072*	
C2C	0.1005 (5)	−0.0353 (4)	0.3093 (3)	0.0705 (14)	
H2C	0.0949	−0.0967	0.3479	0.085*	
C3C	0.0693 (5)	−0.0374 (4)	0.2410 (4)	0.0704 (14)	
H3C	0.0424	−0.1004	0.2323	0.084*	
C4C	0.0774 (4)	0.0547 (4)	0.1836 (3)	0.0570 (11)	
C5C	0.0484 (5)	0.0587 (5)	0.1095 (3)	0.0703 (14)	
H5C	0.0206	−0.0022	0.0982	0.084*	
C6C	0.0606 (5)	0.1494 (5)	0.0558 (3)	0.0718 (15)	
H6C	0.0429	0.1490	0.0075	0.086*	
C7C	0.0999 (4)	0.2462 (4)	0.0706 (2)	0.0591 (12)	
C8C	0.1121 (5)	0.3444 (5)	0.0179 (3)	0.0732 (15)	
H8C	0.0942	0.3494	−0.0310	0.088*	
C9C	0.1498 (5)	0.4320 (5)	0.0378 (3)	0.0720 (14)	
H9C	0.1572	0.4973	0.0030	0.086*	
C10C	0.1774 (5)	0.4233 (4)	0.1108 (2)	0.0576 (11)	
H10C	0.2030	0.4840	0.1239	0.069*	
C11C	0.1301 (4)	0.2445 (4)	0.1436 (2)	0.0458 (9)	
C12C	0.1190 (4)	0.1469 (3)	0.2003 (2)	0.0457 (9)	
O1W	0.5525 (6)	0.4757 (5)	0.5714 (3)	0.1193 (17)	
H1WA	0.547 (10)	0.410 (3)	0.596 (4)	0.179*	
H1WB	0.516 (10)	0.522 (5)	0.604 (4)	0.179*	
S1	0.87231 (13)	0.74846 (10)	0.19276 (6)	0.0597 (3)	
S2	0.51188 (14)	0.84299 (10)	0.32020 (7)	0.0632 (3)	
O1	0.9510 (4)	0.7297 (3)	0.2484 (2)	0.0913 (12)	
O2	0.8815 (4)	0.8464 (3)	0.13880 (19)	0.0892 (12)	
O3	0.8770 (5)	0.6481 (3)	0.1618 (2)	0.0980 (12)	
O4	0.7174 (4)	0.7544 (4)	0.2327 (3)	0.0779 (13)	0.617 (3)
O5	0.6654 (4)	0.8441 (5)	0.2768 (3)	0.0797 (14)	0.617 (3)
O6	0.5143 (6)	0.7390 (3)	0.3684 (2)	0.1142 (16)	
O7	0.5037 (4)	0.9379 (3)	0.3577 (2)	0.0915 (12)	
O8	0.4358 (4)	0.8641 (3)	0.2626 (2)	0.0968 (13)	
N1D	0.7290 (6)	0.1726 (5)	0.4231 (3)	0.0937 (15)	
O1D'	0.5753 (9)	0.3293 (7)	0.3748 (5)	0.106 (4)	0.544 (12)
C1D	0.6338 (8)	0.2719 (7)	0.4278 (5)	0.106 (2)	
H1DD	0.6100	0.2998	0.4750	0.127*	0.544 (12)
H1DA	0.6247	0.2977	0.4765	0.159*	0.456 (12)
H1DB	0.5495	0.2585	0.4233	0.159*	0.456 (12)
H1DC	0.6608	0.3288	0.3869	0.159*	0.456 (12)
O1D"	0.8949 (16)	0.0480 (15)	0.4893 (8)	0.167 (7)	0.456 (12)
C2D	0.7955 (10)	0.1257 (8)	0.4819 (5)	0.128 (3)	
H2DB	0.7639	0.1718	0.5230	0.192*	0.544 (12)
H2DC	0.8898	0.1219	0.4636	0.192*	0.544 (12)
H2DD	0.7796	0.0505	0.5004	0.192*	0.544 (12)
H2DA	0.7581	0.1594	0.5265	0.154*	0.456 (12)
C3D	0.7575 (9)	0.1204 (9)	0.3550 (5)	0.156 (4)	
H3D1	0.7890	0.1720	0.3115	0.235*	
H3D2	0.6777	0.1016	0.3479	0.235*	
H3D3	0.8253	0.0525	0.3594	0.235*	
N1E	0.4039 (7)	0.7569 (6)	0.0434 (4)	0.1133 (18)	
C1E'	0.506 (2)	0.689 (2)	0.0074 (13)	0.166 (9)	0.524 (12)
H1EA	0.5868	0.7140	0.0062	0.250*	0.524 (12)
H1EB	0.4951	0.6906	−0.0443	0.250*	0.524 (12)
H1EC	0.5112	0.6132	0.0339	0.250*	0.524 (12)
C2E'	0.399 (3)	0.8651 (17)	0.0685 (14)	0.173 (8)	0.524 (12)
H2EA	0.4886	0.8776	0.0610	0.259*	0.524 (12)
H2EB	0.3585	0.8633	0.1221	0.259*	0.524 (12)
H2EC	0.3475	0.9255	0.0391	0.259*	0.524 (12)
C3E'	0.265 (2)	0.7430 (19)	0.0550 (11)	0.128 (5)	0.524 (12)
H3EA	0.1969	0.7961	0.0796	0.154*	0.524 (12)
O1E'	0.238 (2)	0.6704 (15)	0.0354 (9)	0.171 (6)	0.524 (12)
C1E"	0.296 (3)	0.826 (3)	0.0685 (15)	0.179 (10)	0.476 (12)
H1ED	0.2207	0.7918	0.0722	0.268*	0.476 (12)
H1EE	0.2879	0.8951	0.0337	0.268*	0.476 (12)
H1EF	0.2989	0.8412	0.1184	0.268*	0.476 (12)
C2E"	0.539 (2)	0.797 (2)	0.0370 (14)	0.164 (8)	0.476 (12)
H2ED	0.5188	0.8739	0.0485	0.246*	0.476 (12)
H2EE	0.5904	0.7939	−0.0143	0.246*	0.476 (12)
H2EF	0.5892	0.7484	0.0729	0.246*	0.476 (12)
C3E"	0.436 (3)	0.6469 (19)	0.0256 (13)	0.122 (5)	0.476 (12)
H3EB	0.5246	0.6116	0.0105	0.146*	0.476 (12)
O1E"	0.356 (3)	0.602 (2)	0.0292 (11)	0.181 (7)	0.476 (12)
O4'	0.7492 (13)	0.8086 (11)	0.2641 (7)	0.100 (4)*	0.383 (3)
O5'	0.650 (2)	0.759 (2)	0.3001 (13)	0.078 (8)*	0.180 (10)
O5"	0.6248 (15)	0.8221 (12)	0.2377 (9)	0.050 (5)*	0.202 (10)

### Atomic displacement parameters (Å^2^)

**Table:**

	*U*^11^	*U*^22^	*U*^33^	*U*^12^	*U*^13^	*U*^23^
Ni1	0.0430 (3)	0.0411 (3)	0.0384 (3)	−0.0093 (2)	−0.0099 (2)	−0.0055 (2)
N1A	0.052 (2)	0.0410 (18)	0.0427 (18)	−0.0085 (15)	−0.0085 (15)	−0.0111 (14)
N2A	0.0477 (19)	0.0378 (18)	0.0443 (18)	−0.0061 (15)	−0.0075 (15)	−0.0042 (14)
C1A	0.065 (3)	0.050 (3)	0.056 (3)	−0.010 (2)	−0.007 (2)	−0.019 (2)
C2A	0.099 (4)	0.045 (3)	0.074 (3)	−0.021 (3)	−0.010 (3)	−0.023 (2)
C3A	0.092 (4)	0.061 (3)	0.064 (3)	−0.042 (3)	−0.013 (3)	−0.012 (2)
C4A	0.066 (3)	0.062 (3)	0.048 (2)	−0.029 (2)	−0.016 (2)	−0.006 (2)
C5A	0.063 (3)	0.092 (4)	0.071 (3)	−0.044 (3)	−0.011 (3)	−0.006 (3)
C6A	0.046 (3)	0.101 (5)	0.083 (4)	−0.027 (3)	−0.005 (3)	−0.005 (3)
C7A	0.045 (2)	0.069 (3)	0.059 (3)	−0.007 (2)	−0.005 (2)	−0.002 (2)
C8A	0.051 (3)	0.077 (4)	0.077 (3)	0.010 (3)	0.002 (2)	−0.006 (3)
C9A	0.068 (3)	0.053 (3)	0.077 (3)	0.014 (3)	−0.003 (3)	−0.009 (2)
C10A	0.064 (3)	0.050 (3)	0.054 (3)	−0.007 (2)	−0.009 (2)	−0.003 (2)
C11A	0.044 (2)	0.053 (2)	0.040 (2)	−0.0096 (19)	−0.0093 (17)	−0.0028 (18)
C12A	0.045 (2)	0.051 (2)	0.039 (2)	−0.0152 (19)	−0.0107 (17)	−0.0042 (17)
N1B	0.0424 (18)	0.0446 (18)	0.0401 (17)	−0.0111 (15)	−0.0093 (14)	0.0034 (14)
N2B	0.0461 (19)	0.0437 (18)	0.0460 (18)	−0.0046 (15)	−0.0145 (15)	−0.0087 (15)
C1B	0.045 (2)	0.066 (3)	0.047 (2)	−0.005 (2)	−0.0110 (19)	0.001 (2)
C2B	0.040 (2)	0.058 (3)	0.070 (3)	−0.003 (2)	−0.010 (2)	0.002 (2)
C3B	0.040 (2)	0.063 (3)	0.062 (3)	−0.007 (2)	0.002 (2)	−0.013 (2)
C4B	0.047 (2)	0.053 (2)	0.048 (2)	−0.018 (2)	0.0004 (18)	−0.0088 (19)
C5B	0.061 (3)	0.080 (3)	0.049 (3)	−0.024 (3)	0.003 (2)	−0.018 (2)
C6B	0.073 (3)	0.071 (3)	0.038 (2)	−0.023 (3)	−0.004 (2)	−0.012 (2)
C7B	0.061 (3)	0.057 (3)	0.040 (2)	−0.018 (2)	−0.013 (2)	−0.0051 (19)
C8B	0.079 (3)	0.080 (3)	0.044 (2)	−0.013 (3)	−0.026 (2)	−0.007 (2)
C9B	0.070 (3)	0.085 (4)	0.061 (3)	−0.001 (3)	−0.034 (3)	−0.006 (3)
C10B	0.053 (3)	0.067 (3)	0.059 (3)	−0.001 (2)	−0.022 (2)	−0.010 (2)
C11B	0.048 (2)	0.043 (2)	0.039 (2)	−0.0168 (18)	−0.0115 (17)	−0.0066 (16)
C12B	0.042 (2)	0.042 (2)	0.041 (2)	−0.0174 (17)	−0.0052 (16)	−0.0070 (16)
N1C	0.0462 (19)	0.053 (2)	0.0458 (19)	−0.0155 (16)	−0.0105 (15)	−0.0049 (16)
N2C	0.0463 (19)	0.051 (2)	0.0401 (17)	−0.0078 (16)	−0.0085 (15)	−0.0060 (15)
C1C	0.070 (3)	0.052 (3)	0.062 (3)	−0.022 (2)	−0.018 (2)	−0.001 (2)
C2C	0.074 (3)	0.052 (3)	0.089 (4)	−0.023 (3)	−0.020 (3)	0.001 (3)
C3C	0.059 (3)	0.060 (3)	0.104 (4)	−0.019 (2)	−0.018 (3)	−0.029 (3)
C4C	0.041 (2)	0.061 (3)	0.074 (3)	−0.010 (2)	−0.010 (2)	−0.026 (2)
C5C	0.059 (3)	0.082 (4)	0.083 (4)	−0.014 (3)	−0.015 (3)	−0.044 (3)
C6C	0.059 (3)	0.102 (4)	0.064 (3)	−0.003 (3)	−0.021 (2)	−0.043 (3)
C7C	0.044 (2)	0.087 (3)	0.047 (2)	0.000 (2)	−0.0112 (19)	−0.026 (2)
C8C	0.067 (3)	0.106 (4)	0.042 (3)	0.001 (3)	−0.019 (2)	−0.013 (3)
C9C	0.078 (3)	0.083 (4)	0.045 (3)	−0.006 (3)	−0.017 (2)	0.010 (3)
C10C	0.062 (3)	0.061 (3)	0.045 (2)	−0.010 (2)	−0.012 (2)	0.002 (2)
C11C	0.0330 (19)	0.061 (3)	0.042 (2)	−0.0020 (18)	−0.0055 (16)	−0.0170 (19)
C12C	0.035 (2)	0.052 (2)	0.052 (2)	−0.0083 (18)	−0.0062 (17)	−0.0164 (19)
O1W	0.096 (3)	0.109 (4)	0.127 (4)	−0.023 (3)	−0.002 (3)	0.029 (3)
S1	0.0785 (8)	0.0605 (7)	0.0471 (6)	−0.0279 (6)	−0.0163 (6)	−0.0020 (5)
S2	0.0782 (8)	0.0452 (6)	0.0671 (7)	−0.0088 (6)	−0.0203 (6)	−0.0065 (5)
O1	0.113 (3)	0.085 (3)	0.096 (3)	−0.028 (2)	−0.059 (2)	−0.002 (2)
O2	0.127 (3)	0.075 (2)	0.067 (2)	−0.039 (2)	−0.019 (2)	0.0123 (17)
O3	0.166 (4)	0.079 (2)	0.066 (2)	−0.047 (2)	−0.032 (2)	−0.0130 (17)
O4	0.085 (2)	0.082 (3)	0.084 (3)	−0.034 (3)	−0.021 (2)	−0.028 (2)
O5	0.080 (2)	0.066 (3)	0.104 (4)	−0.016 (2)	−0.025 (2)	−0.027 (2)
O6	0.182 (5)	0.066 (2)	0.090 (3)	−0.041 (3)	−0.021 (3)	0.0140 (19)
O7	0.132 (3)	0.063 (2)	0.088 (3)	−0.008 (2)	−0.037 (2)	−0.0262 (18)
O8	0.108 (3)	0.062 (2)	0.141 (3)	−0.004 (2)	−0.074 (3)	−0.021 (2)
N1D	0.094 (4)	0.109 (4)	0.084 (3)	−0.041 (3)	−0.013 (3)	−0.008 (3)
O1D'	0.115 (7)	0.096 (6)	0.120 (7)	−0.034 (5)	−0.049 (6)	0.006 (5)
C1D	0.110 (5)	0.100 (5)	0.120 (6)	−0.044 (4)	−0.030 (4)	−0.006 (4)
O1D"	0.152 (12)	0.213 (16)	0.108 (9)	−0.004 (9)	−0.034 (8)	0.019 (9)
C2D	0.146 (7)	0.135 (7)	0.107 (5)	−0.041 (5)	−0.042 (5)	0.013 (5)
C3D	0.122 (7)	0.223 (10)	0.121 (6)	0.005 (7)	−0.018 (5)	−0.076 (7)
N1E	0.117 (5)	0.102 (4)	0.100 (4)	−0.005 (4)	−0.003 (4)	−0.006 (3)
C1E'	0.156 (13)	0.162 (17)	0.126 (14)	0.037 (13)	0.000 (13)	−0.003 (12)
C2E'	0.183 (18)	0.131 (12)	0.206 (18)	−0.065 (12)	0.003 (16)	−0.035 (11)
C3E'	0.139 (9)	0.107 (11)	0.135 (12)	−0.038 (9)	−0.006 (10)	−0.015 (10)
O1E'	0.226 (16)	0.144 (12)	0.152 (11)	−0.085 (12)	−0.001 (12)	−0.029 (9)
C1E"	0.132 (12)	0.175 (18)	0.173 (17)	0.027 (14)	0.015 (15)	−0.017 (17)
C2E"	0.146 (12)	0.138 (15)	0.204 (19)	−0.051 (12)	−0.010 (15)	−0.011 (14)
C3E"	0.146 (14)	0.121 (10)	0.103 (11)	−0.045 (9)	−0.025 (11)	0.001 (10)
O1E"	0.220 (19)	0.204 (15)	0.152 (12)	−0.120 (14)	−0.040 (14)	0.000 (12)

### Geometric parameters (Å, º)

**Table:**

Ni1—N2B	2.088 (3)	C4C—C12C	1.402 (6)
Ni1—N2C	2.089 (3)	C4C—C5C	1.425 (7)
Ni1—N1B	2.090 (3)	C5C—C6C	1.344 (7)
Ni1—N2A	2.091 (3)	C5C—H5C	0.9300
Ni1—N1C	2.098 (3)	C6C—C7C	1.428 (7)
Ni1—N1A	2.100 (3)	C6C—H6C	0.9300
N1A—C1A	1.321 (5)	C7C—C8C	1.403 (7)
N1A—C12A	1.349 (5)	C7C—C11C	1.416 (5)
N2A—C10A	1.347 (5)	C8C—C9C	1.356 (7)
N2A—C11A	1.355 (5)	C8C—H8C	0.9300
C1A—C2A	1.391 (6)	C9C—C10C	1.390 (6)
C1A—H1A	0.9300	C9C—H9C	0.9300
C2A—C3A	1.361 (7)	C10C—H10C	0.9300
C2A—H2A	0.9300	C11C—C12C	1.433 (6)
C3A—C4A	1.399 (7)	O1W—H1WA	0.854 (13)
C3A—H3A	0.9300	O1W—H1WB	0.850 (13)
C4A—C12A	1.400 (6)	S1—O1	1.395 (3)
C4A—C5A	1.428 (7)	S1—O2	1.408 (3)
C5A—C6A	1.330 (8)	S1—O3	1.422 (3)
C5A—H5A	0.9300	S1—O4	1.608 (3)
C6A—C7A	1.439 (7)	S2—O8	1.401 (3)
C6A—H6A	0.9300	S2—O6	1.403 (3)
C7A—C8A	1.384 (7)	S2—O7	1.420 (3)
C7A—C11A	1.412 (6)	S2—O5	1.622 (3)
C8A—C9A	1.362 (7)	O4—O5	1.399 (4)
C8A—H8A	0.9300	N1D—C2D	1.366 (9)
C9A—C10A	1.400 (7)	N1D—C1D	1.377 (9)
C9A—H9A	0.9300	N1D—C3D	1.422 (9)
C10A—H10A	0.9300	O1D'—C1D	1.267 (10)
C11A—C12A	1.433 (6)	C1D—H1DD	0.9300
N1B—C1B	1.332 (5)	C1D—H1DA	0.9600
N1B—C12B	1.357 (5)	C1D—H1DB	0.9600
N2B—C10B	1.326 (5)	C1D—H1DC	0.9600
N2B—C11B	1.354 (5)	O1D"—C2D	1.246 (15)
C1B—C2B	1.383 (6)	C2D—H2DB	0.9600
C1B—H1B	0.9300	C2D—H2DC	0.9600
C2B—C3B	1.362 (6)	C2D—H2DD	0.9600
C2B—H2B	0.9300	C2D—H2DA	0.9300
C3B—C4B	1.403 (6)	C3D—H3D1	0.9600
C3B—H3B	0.9300	C3D—H3D2	0.9600
C4B—C12B	1.395 (5)	C3D—H3D3	0.9600
C4B—C5B	1.431 (6)	N1E—C1E"	1.28 (2)
C5B—C6B	1.360 (7)	N1E—C1E'	1.30 (2)
C5B—H5B	0.9300	N1E—C3E"	1.38 (2)
C6B—C7B	1.427 (6)	N1E—C2E'	1.46 (2)
C6B—H6B	0.9300	N1E—C3E'	1.47 (2)
C7B—C11B	1.395 (5)	N1E—C2E"	1.58 (2)
C7B—C8B	1.400 (6)	C1E'—H1EA	0.9600
C8B—C9B	1.354 (7)	C1E'—H1EB	0.9600
C8B—H8B	0.9300	C1E'—H1EC	0.9600
C9B—C10B	1.397 (6)	C2E'—H2EA	0.9600
C9B—H9B	0.9300	C2E'—H2EB	0.9600
C10B—H10B	0.9300	C2E'—H2EC	0.9600
C11B—C12B	1.449 (5)	C3E'—O1E'	1.13 (2)
N1C—C1C	1.322 (5)	C3E'—H3EA	0.9300
N1C—C12C	1.354 (5)	C1E"—H1ED	0.9600
N2C—C10C	1.326 (5)	C1E"—H1EE	0.9600
N2C—C11C	1.349 (5)	C1E"—H1EF	0.9600
C1C—C2C	1.398 (6)	C2E"—H2ED	0.9600
C1C—H1C	0.9300	C2E"—H2EE	0.9600
C2C—C3C	1.350 (7)	C2E"—H2EF	0.9600
C2C—H2C	0.9300	C3E"—O1E"	1.09 (2)
C3C—C4C	1.390 (7)	C3E"—H3EB	0.9300
C3C—H3C	0.9300		
			
N2B—Ni1—N2C	170.47 (12)	C3C—C4C—C5C	123.6 (4)
N2B—Ni1—N1B	79.71 (12)	C12C—C4C—C5C	119.3 (5)
N2C—Ni1—N1B	94.79 (12)	C6C—C5C—C4C	120.8 (5)
N2B—Ni1—N2A	96.45 (13)	C6C—C5C—H5C	119.6
N2C—Ni1—N2A	89.93 (13)	C4C—C5C—H5C	119.6
N1B—Ni1—N2A	172.08 (12)	C5C—C6C—C7C	122.0 (4)
N2B—Ni1—N1C	92.80 (13)	C5C—C6C—H6C	119.0
N2C—Ni1—N1C	79.70 (13)	C7C—C6C—H6C	119.0
N1B—Ni1—N1C	93.56 (13)	C8C—C7C—C11C	116.6 (4)
N2A—Ni1—N1C	93.54 (13)	C8C—C7C—C6C	125.0 (4)
N2B—Ni1—N1A	94.40 (12)	C11C—C7C—C6C	118.5 (5)
N2C—Ni1—N1A	93.77 (13)	C9C—C8C—C7C	120.3 (4)
N1B—Ni1—N1A	94.29 (13)	C9C—C8C—H8C	119.9
N2A—Ni1—N1A	79.02 (13)	C7C—C8C—H8C	119.9
N1C—Ni1—N1A	170.17 (13)	C8C—C9C—C10C	119.4 (5)
C1A—N1A—C12A	117.9 (4)	C8C—C9C—H9C	120.3
C1A—N1A—Ni1	128.8 (3)	C10C—C9C—H9C	120.3
C12A—N1A—Ni1	113.3 (3)	N2C—C10C—C9C	122.6 (5)
C10A—N2A—C11A	117.9 (4)	N2C—C10C—H10C	118.7
C10A—N2A—Ni1	128.7 (3)	C9C—C10C—H10C	118.7
C11A—N2A—Ni1	113.4 (3)	N2C—C11C—C7C	122.5 (4)
N1A—C1A—C2A	122.4 (5)	N2C—C11C—C12C	118.2 (3)
N1A—C1A—H1A	118.8	C7C—C11C—C12C	119.4 (4)
C2A—C1A—H1A	118.8	N1C—C12C—C4C	123.0 (4)
C3A—C2A—C1A	119.8 (5)	N1C—C12C—C11C	116.9 (3)
C3A—C2A—H2A	120.1	C4C—C12C—C11C	120.1 (4)
C1A—C2A—H2A	120.1	H1WA—O1W—H1WB	105 (3)
C2A—C3A—C4A	119.6 (4)	O1—S1—O2	115.5 (2)
C2A—C3A—H3A	120.2	O1—S1—O3	112.9 (3)
C4A—C3A—H3A	120.2	O2—S1—O3	115.6 (2)
C12A—C4A—C3A	116.5 (4)	O1—S1—O4	110.5 (3)
C12A—C4A—C5A	119.4 (4)	O2—S1—O4	108.3 (3)
C3A—C4A—C5A	124.1 (4)	O3—S1—O4	91.0 (2)
C6A—C5A—C4A	121.5 (4)	O8—S2—O6	117.0 (3)
C6A—C5A—H5A	119.2	O8—S2—O7	115.2 (2)
C4A—C5A—H5A	119.2	O6—S2—O7	115.1 (3)
C5A—C6A—C7A	121.6 (5)	O8—S2—O5	106.7 (3)
C5A—C6A—H6A	119.2	O6—S2—O5	106.5 (3)
C7A—C6A—H6A	119.2	O7—S2—O5	92.5 (2)
C8A—C7A—C11A	117.4 (4)	O5—O4—S1	112.6 (3)
C8A—C7A—C6A	124.6 (5)	O4—O5—S2	111.3 (3)
C11A—C7A—C6A	117.9 (5)	C2D—N1D—C1D	121.1 (7)
C9A—C8A—C7A	121.0 (5)	C2D—N1D—C3D	120.7 (8)
C9A—C8A—H8A	119.5	C1D—N1D—C3D	118.2 (7)
C7A—C8A—H8A	119.5	O1D'—C1D—N1D	127.0 (9)
C8A—C9A—C10A	118.5 (5)	O1D'—C1D—H1DD	116.5
C8A—C9A—H9A	120.7	N1D—C1D—H1DD	116.5
C10A—C9A—H9A	120.7	O1D'—C1D—H1DA	123.2
N2A—C10A—C9A	122.7 (4)	N1D—C1D—H1DA	109.5
N2A—C10A—H10A	118.6	N1D—C1D—H1DB	109.5
C9A—C10A—H10A	118.6	H1DD—C1D—H1DB	100.5
N2A—C11A—C7A	122.5 (4)	H1DA—C1D—H1DB	109.5
N2A—C11A—C12A	117.1 (4)	N1D—C1D—H1DC	109.5
C7A—C11A—C12A	120.4 (4)	H1DD—C1D—H1DC	110.9
N1A—C12A—C4A	123.7 (4)	H1DA—C1D—H1DC	109.5
N1A—C12A—C11A	117.2 (3)	H1DB—C1D—H1DC	109.5
C4A—C12A—C11A	119.1 (4)	O1D"—C2D—N1D	134.5 (12)
C1B—N1B—C12B	116.9 (4)	O1D"—C2D—H2DB	115.3
C1B—N1B—Ni1	129.8 (3)	N1D—C2D—H2DB	109.5
C12B—N1B—Ni1	113.2 (2)	N1D—C2D—H2DC	109.5
C10B—N2B—C11B	117.8 (4)	H2DB—C2D—H2DC	109.5
C10B—N2B—Ni1	129.0 (3)	N1D—C2D—H2DD	109.5
C11B—N2B—Ni1	113.2 (2)	H2DB—C2D—H2DD	109.5
N1B—C1B—C2B	123.7 (4)	H2DC—C2D—H2DD	109.5
N1B—C1B—H1B	118.2	O1D"—C2D—H2DA	112.8
C2B—C1B—H1B	118.2	N1D—C2D—H2DA	112.8
C3B—C2B—C1B	119.3 (4)	H2DC—C2D—H2DA	115.8
C3B—C2B—H2B	120.3	H2DD—C2D—H2DA	99.3
C1B—C2B—H2B	120.3	N1D—C3D—H3D1	109.5
C2B—C3B—C4B	119.3 (4)	N1D—C3D—H3D2	109.5
C2B—C3B—H3B	120.3	H3D1—C3D—H3D2	109.5
C4B—C3B—H3B	120.3	N1D—C3D—H3D3	109.5
C12B—C4B—C3B	117.4 (4)	H3D1—C3D—H3D3	109.5
C12B—C4B—C5B	119.2 (4)	H3D2—C3D—H3D3	109.5
C3B—C4B—C5B	123.3 (4)	C1E"—N1E—C1E'	170.5 (19)
C6B—C5B—C4B	120.7 (4)	C1E"—N1E—C3E"	135 (2)
C6B—C5B—H5B	119.6	C1E'—N1E—C2E'	128.9 (19)
C4B—C5B—H5B	119.6	C3E"—N1E—C2E'	166.9 (17)
C5B—C6B—C7B	121.3 (4)	C1E'—N1E—C3E'	123.3 (18)
C5B—C6B—H6B	119.4	C3E"—N1E—C3E'	83.9 (15)
C7B—C6B—H6B	119.4	C2E'—N1E—C3E'	107.2 (14)
C11B—C7B—C8B	117.0 (4)	C1E"—N1E—C2E"	116.1 (19)
C11B—C7B—C6B	119.3 (4)	C1E'—N1E—C2E"	68.7 (15)
C8B—C7B—C6B	123.7 (4)	C3E"—N1E—C2E"	108.3 (15)
C9B—C8B—C7B	119.8 (4)	C3E'—N1E—C2E"	167.8 (14)
C9B—C8B—H8B	120.1	N1E—C1E'—H1EA	109.5
C7B—C8B—H8B	120.1	N1E—C1E'—H1EB	109.5
C8B—C9B—C10B	119.6 (5)	H1EA—C1E'—H1EB	109.5
C8B—C9B—H9B	120.2	N1E—C1E'—H1EC	109.5
C10B—C9B—H9B	120.2	H1EA—C1E'—H1EC	109.5
N2B—C10B—C9B	122.4 (5)	H1EB—C1E'—H1EC	109.5
N2B—C10B—H10B	118.8	N1E—C2E'—H2EA	109.5
C9B—C10B—H10B	118.8	N1E—C2E'—H2EB	109.5
N2B—C11B—C7B	123.4 (4)	H2EA—C2E'—H2EB	109.5
N2B—C11B—C12B	117.0 (3)	N1E—C2E'—H2EC	109.5
C7B—C11B—C12B	119.6 (4)	H2EA—C2E'—H2EC	109.5
N1B—C12B—C4B	123.3 (4)	H2EB—C2E'—H2EC	109.5
N1B—C12B—C11B	116.9 (3)	O1E'—C3E'—N1E	123 (2)
C4B—C12B—C11B	119.9 (3)	O1E'—C3E'—H3EA	118.7
C1C—N1C—C12C	117.7 (4)	N1E—C3E'—H3EA	118.7
C1C—N1C—Ni1	129.6 (3)	N1E—C1E"—H1ED	109.5
C12C—N1C—Ni1	112.6 (3)	N1E—C1E"—H1EE	109.5
C10C—N2C—C11C	118.7 (3)	H1ED—C1E"—H1EE	109.5
C10C—N2C—Ni1	128.9 (3)	N1E—C1E"—H1EF	109.5
C11C—N2C—Ni1	112.4 (3)	H1ED—C1E"—H1EF	109.5
N1C—C1C—C2C	122.6 (4)	H1EE—C1E"—H1EF	109.5
N1C—C1C—H1C	118.7	N1E—C2E"—H2ED	109.5
C2C—C1C—H1C	118.7	N1E—C2E"—H2EE	109.5
C3C—C2C—C1C	119.6 (5)	H2ED—C2E"—H2EE	109.5
C3C—C2C—H2C	120.2	N1E—C2E"—H2EF	109.5
C1C—C2C—H2C	120.2	H2ED—C2E"—H2EF	109.5
C2C—C3C—C4C	120.0 (4)	H2EE—C2E"—H2EF	109.5
C2C—C3C—H3C	120.0	O1E"—C3E"—N1E	120 (3)
C4C—C3C—H3C	120.0	O1E"—C3E"—H3EB	120.0
C3C—C4C—C12C	117.1 (4)	N1E—C3E"—H3EB	120.0

### Hydrogen-bond geometry (Å, º)

**Table:**

*D*—H···*A*	*D*—H	H···*A*	*D*···*A*	*D*—H···*A*
O1*W*—H1*WA*···O6^i^	0.85 (5)	2.02 (6)	2.839 (7)	160 (10)
O1*W*—H1*WB*···O1*D*′^i^	0.85 (7)	1.90 (7)	2.668 (10)	149 (7)
C3*B*—H3*B*···O1*W*^ii^	0.93	2.54	3.305 (8)	139
C1*B*—H1*B*···O3^ii^	0.93	2.55	3.192 (6)	126
C3*A*—H3*A*···O8	0.93	2.59	3.271 (6)	130
C3*C*—H3*C*···O1^iii^	0.93	2.43	3.337 (6)	164
C5*A*—H5*A*···O3	0.93	2.58	3.505 (7)	170
C5*C*—H5*C*···O2^iii^	0.93	2.53	3.365 (7)	150
C6*B*—H6*B*···O1^i^	0.93	2.53	3.434 (5)	163
C6*C*—H6*C*···O2^iv^	0.93	2.56	3.409 (6)	151
C8*C*—H8*C*···O3^iv^	0.93	2.30	3.197 (6)	162
C10*A*—H10*A*···O8^v^	0.93	2.48	3.220 (6)	137
C10*C*—H10*C*···O1*E*′	0.93	2.59	3.228 (19)	126

Symmetry codes: (i) −*x*+1, −*y*+1, −*z*+1; (ii) *x*−1, *y*, *z*; (iii) *x*−1, *y*−1, *z*; (iv) −*x*+1, −*y*+1, −*z*; (v) *x*, *y*−1, *z*.
